# Long‐term subtropical grassland plots take a long time to change: Replacement is more important than richness differences for beta diversity

**DOI:** 10.1002/ece3.10195

**Published:** 2023-06-14

**Authors:** David Ward, Kevin Kirkman, Craig Morris

**Affiliations:** ^1^ Department of Biological Sciences Kent State University Kent Ohio USA; ^2^ School of Life Sciences University of KwaZulu‐Natal Scottsville South Africa; ^3^ Agricultural Research Council – Animal Production c/o University of KwaZulu‐Natal Pietermaritzburg South Africa

**Keywords:** burning, community composition, community ecology, grass species contributions to beta diversity, local contributions to beta diversity, mowing, replacement, richness differences, subtropical grasslands

## Abstract

We studied β diversity of grasses in a subtropical grassland over 60 years in South Africa. We examined the effects of burning and mowing on 132 large plots. We sought to determine the effects of burning and mowing, and mowing frequency, on the replacement of species and the species richness. We conducted the study at Ukulinga, research farm of the University of KwaZulu‐Natal, Pietermaritzburg, South Africa (29°24′E, 30°24′S) from 1950–2010. Plots were burned annually, biennially, triennially, and a control (unburned). Plots were mowed in spring, late summer, spring plus late summer, and a control (unmowed). We calculated β diversity, with a focus on replacement and richness differences. We also used distance‐based redundancy analyses to examine the relative effects of replacement and richness differences on mowing and burning. We used beta regressions to test for the effect of soil depth and its interactions with mowing and burning. There was no significant change in grass beta diversity until 1995. Thereafter, there were changes in β diversity that demonstrated the primary effects of summer mowing frequency. There was no significant effect of richness differences but a strong effect of replacement post‐1995. There was a significant interaction between mowing frequency and soil depth in one of the analyses. Changes in grassland composition took a long time to manifest themselves and were unapparent prior to 1988. However, there was a change in sampling strategy prior to 1988, from point hits to nearest plants, that may also have influenced the rates of changes in replacement and richness differences. Using β‐diversity indices, we found that mowing was more important than burning that burning frequency was unimportant, and there was a significant interaction effect between mowing and soil depth in one of the analyses.

## Graphical abstract

We studied β diversity of grasses in a subtropical grassland over 60 years with different burning and mowing frequencies. Changes in grassland composition took a long time to manifest themselves. We found that summer mowing was the primary driver and that there was a significant interaction effect between mowing and soil depth.

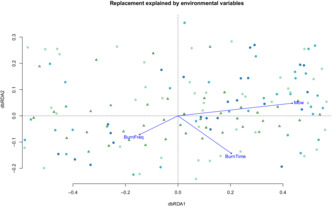

## 1 INTRODUCTION

Grasslands are employed for a variety of land uses, including grazing and hay production (Cavender‐Bares & Reich, 2012; Everson et al., 2021; Fynn et al., 2004; Morris & Fynn, 2001). Many grassland managers use mowing, grazing, burning, and fertilization to improve grazing and hay production (Braun et al., 2022; Cavender‐Bares & Reich, 2012; Chen et al., 2021; Zhu et al., 2021). The variation in plant community structure, as measured by β diversity, is an effective way to assess the importance of controlling factors, such as grazing, mowing, and burning, on biodiversity (Whittaker, 1960, 1965, 1972; Wilsey et al., 2005; Yuan et al., 2016). Mori et al. (2018) consider β diversity to be more effective in determining the processes behind biodiversity change and ecosystem functioning than other indices such as species richness; it provides unique insights into the mechanisms behind changes in biodiversity, particularly under situations that undergo changes caused by disturbances such as effects of burning, mowing, grazing, and fertilization (Fynn et al., 2005).

An earlier study by us (Ward et al., 2020) assessed the similarities and differences in α diversity between a subtropical grassland in South Africa (Ukulinga) and two temperate grasslands (Konza Prairie, USA; Collins et al., 1998) and Park Grass (England; Crawley et al., 2005; Silvertown et al., 2006; Storkey et al., 2016; Tilman et al., 1994). Treatments in the Ukulinga experiment were designed to determine the yield and quality of hay in moist tall grassland due to removal by burning or mowing. We found that there was a strong effect of competition (Ward et al., 2020; Ward, Kirkman, Hagenah, & Tzvuura, 2017; Ward, Kirkman, & Tsvuura, 2017), suggesting that niche‐based assembly rules were dominant (HilleRisLambers et al., 2012). While the field of research based on assembly rules is rich and growing, most of the existing tests are done over very short time scales, which by definition cannot take into account processes such as priority effects (i.e., stochastic variation in species presence and abundance—Kardol et al., 2013; McNaughton, 1983; Uricchio et al., 2019) and competitive exclusion (HilleRisLambers et al., 2012; Ward et al., 2020), which operate over long time spans. In this study, we use a long‐term experimental study (1950–2010) of the native subtropical grassland (Ukulinga) to minimize the problem that priority effects occur. There are remarkably few data that compare subtropical and temperate grasslands (Blair et al., 2014; Gibson, 2009; Ward et al., 2020; Wilsey, 2018). In general, however, subtropical grasslands respond more quickly than temperate grasslands because of higher temperatures and higher rainfall (Leriche et al., 2003). A further advantage of the abovementioned Ukulinga experiment is that there was replication within an appropriate statistical design (Morris & Fynn, 2001; Ward et al., 2020; Ward, Kirkman, Hagenah, & Tzvuura, 2017; Ward, Kirkman, & Tsvuura, 2017), allowing us to test the effects of different manipulative conditions on these grasslands (Fynn et al., 2005; Kirkman et al., 2014). We were particularly interested in the importance of β diversity on three key manipulations (mowing frequency, burning frequency, and burn time), as well as the duration of changes in the grasslands.

Many studies have shown that growth rates of plants affect the rates of change of β diversity; trees grow more slowly than grasses, and thus the rate of change of β diversity should be correspondingly slower in forests (Condit et al., 2002; Duivenvoorden, 1995; Duivenvoorden et al., 2002; Reu et al., 2022; Siefert et al., 2013) than in grasslands (Crawley et al., 2005; Dembicz et al., 2021; Du et al., 2022; Silvertown et al., 2006). Similarly, low nutrient availability, such as in deserts, could also cause diminished rates of change in β diversity (Ward, 2016; Ward et al., 1993). In some grasslands, changes in species composition are reputed to be rapid (Avolio et al., 2014; Balogianni et al., 2014; Ceballos et al., 2010; Morris et al., 1992, 2021; Török et al., 2010). However, more recently, Weisser et al. (2017) and Seabloom et al. (2020) have found that changes in grasslands, at least in temperate grasslands, may be slower than expected.

The division of β diversity into the replacement of certain species and species richness differences is a fundamental advance in our understanding of differences among communities as being more than simply the difference between α (local) diversity and γ diversity (species pools; Ellison, 2010; Legendre & De Cáceres, 2013; Whittaker, 1960, 1965; Zobel, 1997) in terms of products of species change (Schmera & Podani, 2011; Whittaker et al., 2022; Williams, 1996). Replacement is called turnover when analyzed along spatial or environmental gradients (Legendre, 2014) or is due to variation in community composition among sampling plots but does not necessarily recognize explicit gradients such as occurs in applied ecological settings (Anderson et al., 2011; Vellend, 2001). Species richness differences, on the other hand, may result from the diversity of niches available in different locations (Carvalho et al., 2012). Richness differences can be caused by species loss or gain along a particular environment gradient (Fynn et al., 2011), or richness differences may be caused by physical barriers or historical events (Carvalho et al., 2013; Godsoe et al., 2022; Leprieur et al., 2012). Such richness differences may occur because certain species are more tolerant (or less so) of a wide range of environments than others, or richness differences may be caused by differential colonization or extinction rates or may result from dispersal limitations (Novotny & Weiblen, 2005). In the extreme form, species richness differences may be nested or ordered subsets of one another (Almeida‐Neto et al., 2012; Atmar & Patterson, 1993; Baselga, 2012).

In this study, we use β diversity measures of grass species replacement and richness differences (Legendre, 2014) to investigate the effects of intensification of land use for hay production that may be achieved by mowing and burning in 132 plots, each of 18.3 m × 13.7 m (20 yards × 15 yards) that were adjacent to each other (Ward et al., 2020; Ward, Kirkman, Hagenah, & Tzvuura, 2017; Ward, Kirkman, & Tsvuura, 2017). We used annual, biennial, and triennial burning. Mowing mostly occurred once a year in early or late summer, or twice a year at the same time periods. We investigated the effects of summer mowing and burning at different frequencies relative to β diversity effects over time, roughly at 10‐year intervals (Ward, Kirkman, Hagenah, & Tzvuura, 2017). We did not have control over the frequency of the sampling but believe that more frequent measurements could have led to more trivial results (e.g., comparing annual or biennial sampling may not have shown differences, but comparing every 10 years or so could have shown us differences). Soil depth may also affect β diversity (Braun et al., 2022). There is little diversity in altitude (838–847 m) in a relatively low topographic gradient at Ukulinga, but there was considerable variability in soil depth. We had data on mean soil depth in all 132 plots as well as at a gross scale, where there were two types of soils, Mispah (shallow soils) and Westleigh (deeper soils; Soil Classification Working Group, 1991). It is frequently assumed that there will be increased β diversity on deeper soils because they contain more species than shallow soils by supplying space for increased belowground niche partitioning (e.g., Baer et al., 2016; Belcher et al., 1995; Braun et al., 2022; Martorell et al., 2015). However, β diversity may decline through trade‐offs for a few highly productive species, leading to reduced diversity on deep soils (Abrams & Hulbert, 1987; Braun et al., 2022; Fry et al., 2021; Gibson & Hulbert, 1987).

We made the following predictions:

1. Most variance in grass β diversity will be due to replacement such as created by environmental filtering (e.g., between mowing and burning levels) and/or niche partitioning (species outcompete each other because of dominance under certain environmental conditions—Adler et al., 2013; Godsoe et al., 2022) rather than richness differences (Seabloom et al., 2020).
2. The process of grassland change will be rapid due to relatively high precipitation and temperature (resulting in high productivity).
3. Grass β diversity will change more rapidly with burning than with mowing (Collins et al., 1998; Ward, Kirkman, Hagenah, & Tzvuura, 2017).
4. Soil depth may also affect β diversity due to greater belowground niche partitioning, but it may be reduced by disturbances such as fire and mowing (Braun et al., 2022).

## 2 METHODS

The vegetation of the area is classified at a large spatial scale as KwaZulu‐Natal hinterland thornveld (Mucina & Rutherford, 2006), which is an open savanna of *Vachellia* (Syn. *Acacia*) *sieberiana* (Burtt Davy) Kyal. & Boatwr. Trees and *Hyparrhenia hirta* (L.) Stapf, *Aristida junciformis* Trin. & Rupr., *Themeda triandra* Forssk. and other grass species. At a smaller spatial scale (as on the escarpment of Ukulinga), the vegetation is tall grassveld (Acocks, 1953). With regular burning, trees are sparse and *T. triandra* is the dominant grass, with *Tristachya leucothrix* Trin. Ex Nees and *Heteropogon contortus* (L.) Roem. & Schult. also being common (Morris & Fynn, 2001; Ward et al., 2020; Ward, Kirkman, Hagenah, & Tzvuura, 2017; Ward, Kirkman, & Tsvuura, 2017). The native grass species in the locality all use the C_4_ photosynthetic pathway and are all perennials (Fynn et al., 2004). We consulted Tainton et al. (1976) and Van Oudtshoorn (2012) for grass identification. We used Fish et al. (2015) to resolve all grass species' names. Together, these grass species account for most of the herbaceous aboveground net primary production (ANPP) at Ukulinga (Fynn et al., 2004). All forbs constitute <10% of the vegetation biomass in any given census (Fynn et al., 2004; Uys et al., 2004; Ward et al., 2020; Ward, Kirkman, Hagenah, & Tzvuura, 2017). For the census intervals, mean forb ± SE relative abundance was low (6.08 ± 1.30%; Ward et al., 2020). We collected data on the species composition of the forbs in 2010 but not prior to that. The focus of this experiment was strongly linked to hay production, and forbs did not respond consistently, and contributed little to aerial or basal cover (Fynn et al., 2004; Ward et al., 2020). Sampling was done at the peak of the growing season, and samples were spread evenly across the plots. For the first three surveys (1955, 1968, 1975), the method counted basal cover hits on species by descending points (*n* = 200) in a point‐intercept frame (Jonasson, 1988), also known as a Levy bridge frame (Levy & Madden, 1933; Mueller‐Dombois & Ellenberg, 1974). In later surveys, the nearest‐plant point method was used for assessing species composition (Hardy & Tainton, 1993; Mentis, 1981; Mentis et al., 1980; Short & Morris, 2016), with 200 sample points taken per treatment plot in 1988 (Fynn et al., 2004), and 100 sample points taken per treatment plot in 1988 and 2010. Thus, the method of assessing the Ukulinga long‐term experiments was changed in the 1980s to the nearest‐plant frequency method, usually using a metal spike to locate points (Fynn et al., 2004; Owensby, 1973; Short & Morris, 2016).

The mean annual precipitation in the locality is 790 mm (32‐year mean), about 80% of which falls during summer as convective storms (October–April). Mean monthly maximum and minimum temperatures range from 26.4°C in February to 8.8°C in July, respectively. Winters are mild with a mean maximum of 13.2°C in July (43‐year mean), with occasional frost. There has been no grazing on the experimental sites for >70 years. The experiment was situated on top of a small escarpment, ranging in altitude between 838 and 847 m. Soils are fine‐textured and derived from shales and were classified as Mispah (shallow soils) and Westleigh (deeper soils; Soil Classification Working Group, 1991) or plinthic acrisols (FAO, 2001).

### 2.1 Burning and mowing experiment (BME)

**FIGURE 1:**
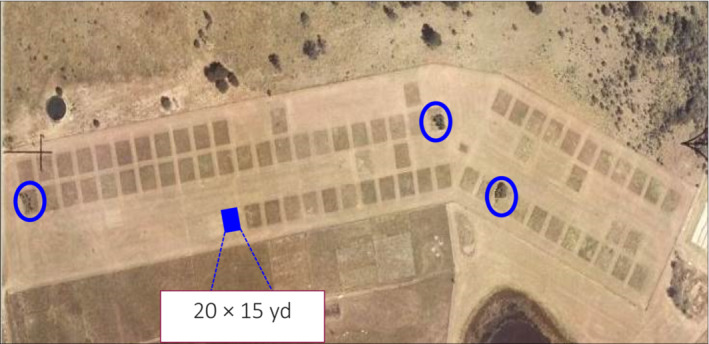
Aerial photograph of the study site. Each plot was 15 yards by 20 yards in size. The complete control plots are indicated in circles, and contain some trees (dark shapes).

**FIGURE 2:**
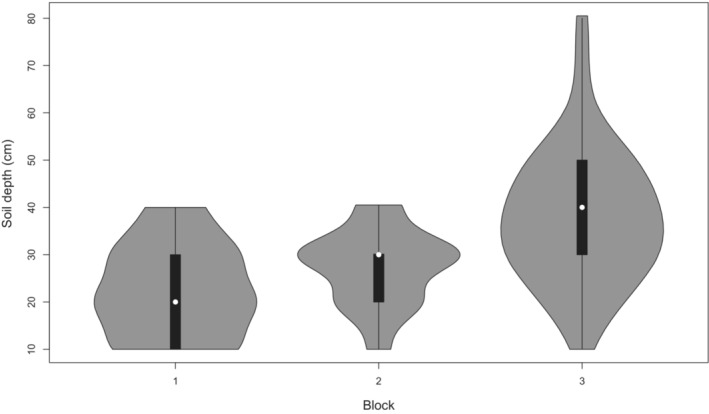
Violin plots of the soil depth (cm) in the three blocks (each block contained 88 plots). There was considerably more variance in soil depth in Block 3 than in Blocks 1 and 2. Medians are indicated in white circles, upper and lower quartiles are indicated in black bars, and the violin is in gray.

A full description of the BME, which was established in 1950 on virgin native grassland is given elsewhere (see table 1 in Fynn et al. (2004) for details; Table S1). The experiment is a split‐plot design in three blocks with four whole‐plot treatments and 11 subplot‐ (18.3 × 13.7 m) fire or mowing treatments separated by rows 4.5 m wide (Morris & Fynn, 2001; Figure 1). Thus, each whole‐plot treatment in a particular block consisted of a row of 11 subplots to which subplot treatments were randomly allocated (Table S1). Whole‐plot treatments were randomly allocated to one of four rows per block. The whole‐plot treatments were: none (no mowing), early summer mowing (December), late summer mowing (February), and early plus late summer mowing. Subplot treatments were: no burning (control), annual, biennial, or triennial burning in winter, spring, or autumn (no annual autumn burn), and annual mowing in winter or spring (the two last‐mentioned were burn‐substitution treatments; Table S1). Burns were done in autumn (mid‐May), first week of August, and in spring after the first effective rains (12.5 mm in 24 h; Table S1). Burn times were recorded in months. Disturbance frequency ranged from plots mown twice in summer and burnt or mown in the dormant period each year to plots completely protected from disturbance for more than 70 years. Soil depth was recorded in each plot because the three blocks did not reflect the large differences in block 3 very effectively (Figure 2). We measured the average soil depth in each of the 132 plots, established by augering to bedrock.

### 2.2 Statistics

We used four beta‐diversity indices. Two of these indices were presence–absence (qualitative; Jaccard and Sørensen) and the other two were quantitative (Ružička and percentage difference). The Jaccard index (Jaccard, 1902, 1912) takes a global view and compares the number of shared species to the *total* number of species whereas the Sørensen index (Sørensen, 1948) takes a local view, comparing the number of shared species to the *mean* number of species in a single assemblage (Jost, 2007; Jost et al., 2011). The Ružička (1958) and percentage difference indices (the latter is also known as Bray–Curtis index; Bray & Curtis, 1957; Gauch, 1973) are quantitative versions of Jaccard and Sørensen, respectively (Legendre, 2014; Podani & Schmera, 2011). Following Borcard et al. (2018), we used absolute differences for the Ružička and percentage differences and richness differences for Jaccard and Sørensen indices. To minimize Type I errors, we used a Holm's (1979) correction of α, with 9999 randomizations (Borcard et al., 2018). We used the software and programming language R version 3.4.3 (R Core Team, 2018) in R Studio (R Studio Team, 2016) for all analyses. All statistical tests used *ade4*, *adespatial* (Borcard et al., 2018), and *vegan* (Oksanen et al., 2020).

We primarily used a two‐dimensional (2D) simplex diagram (Podani & Schmera, 2011) to assess β diversity, and to link it to replacement and richness differences (Borcard et al., 2018; Carvalho et al., 2012; Legendre, 2014). This is an equilateral triangle or ternary plot (Howarth, 1996) commonly applied in the sciences to express the relationship among three quantities.

We calculated the ecological uniqueness of sites, recorded as effects of plots to β diversity (LCBD; Legendre & De Cáceres, 2013). Additionally, we estimated the contributions of species (SCBD) to β diversity across time (Legendre, 2014). Richness difference (LCBD_rich_) and replacement (LCBD_repl_) consider the effects of each individual sample to richness and replacement gradients (Ruhì et al., 2017). We used a Hellinger transformation of the plot‐by‐species presence–absence (qualitative) or abundance (quantitative) community matrix and subsequently calculated the total beta diversity (BD total), LCBD value for each plot, and SCBD value for each species (Legendre & De Cáceres, 2013). We computed the local contributions to β diversity (LCBD) with the *LCBD.comp* function in R (Legendre, 2014; Legendre & De Cáceres, 2013; Ruhì et al., 2017). The difference between presence–absence and abundance‐based partitioning of total β diversity is that Hellinger transformation is based on total species richness and total abundance in each sampling unit (Da Silva & Hernández, 2014). The differences in these values for each species in a given site provide two different values of total β diversity because the sums of squares are based on different means (Da Silva & Hernández, 2014; Vilmi et al., 2017). Different values of total β diversity imply differences in SCBD and LCBD values for both presence–absence and abundance‐based approaches, justifying the assessment of differences between quantitative and qualitative data (Da Silva & Hernández, 2014). Mostly, we recorded the ecological uniqueness of plots (i.e., LCBD; Da Silva et al., 2018) because of our interest in the effects of mowing and burning on β diversity in this grassland (Fynn et al., 2011; Kirkman et al., 2014; Tsvuura & Kirkman, 2013; Ward et al., 2020; Ward, Kirkman, Hagenah, & Tzvuura, 2017; Ward, Kirkman, & Tsvuura, 2017). We were also interested in determining the most important species using SCBD, following Legendre (2014).

Bennett and Gilbert (2015) have also found that the use of both classical and multivariate methods may be effective ways to assess support for particular variables. We used two models to assess the relative importance of the three explanatory variables (mowing, burn frequency, and burn time). The first model was beta regression that examined the same three explanatory variables in 2010 (Da Silva et al., 2018; Ferrari & Cribari‐Neto, 2004; Lindholm et al., 2021). This technique assumes that the dependent variable (LCBD_repl_) is beta distributed, varying between 0 and 1 (Da Silva et al., 2018; Ferrari & Cribari‐Neto, 2004; Lindholm et al., 2021), and that the mean is related to a set of predictor variables (Mowing Frequency (0, 1, 2), Burn Time (expressed in months), and Burn Frequency (0, 1, 2, 3 fire return interval)) via a linear predictor with unknown coefficients and a logit‐link function (Cribari‐Neto & Zeileis, 2010; Douma & Weedon, 2019; Heino & Grönroos, 2017). We used both the Jaccard and percentage difference (Bray–Curtis) indices. We fitted the model using *betareg* from the *betareg* package in R (Cribari‐Neto & Zeileis, 2010; Petsch et al., 2021). The beta regression approach includes features such as heteroskedasticity or skewness, which are typically observed in explanatory data taking values from 0 to 1 (Da Silva et al., 2018). We compared the main effects and the main effects + interaction effects using a likelihood ratio test, *lrtest*, in the package *lmtest* (Hothorn et al., 2022).

The second model was a multivariate canonical ordination, distance‐based redundancy analysis (dbRDA; Borcard et al., 2018; Legendre & Anderson, 1999; McArdle & Anderson, 2001) to test for the relative effects of burning frequency, burning time, and mowing. We used percentage difference (Bray–Curtis) for this index, as recommended by Borcard et al. (2018). We ran a dbRDA triplot to show the changes in community composition in 2010 (Angeler et al., 2009; Bennett & Gilbert, 2015; Legendre & Salvat, 2015). Jupke and Schäfer (2020) have shown that dbRDA had the lowest false‐negative rate of alternative multivariate analyses (see also Schroeder & Jenkins, 2018). Thereafter, the dissimilarities were square‐root transformed to convert it into a Euclidean matrix (Gower, 1985), before computing the entire matrix into a principal coordinate analysis (pCoA; Gower, 1966). We used richness difference and replacement matrices as the explanatory data in an RDA against an environmental matrix representing mowing frequency, burn frequency, and burn time, following Borcard et al. (2018). We plotted the dbRDA using *vegan* (Oksanen et al., 2020).

## 3 RESULTS

**FIGURE 3:**
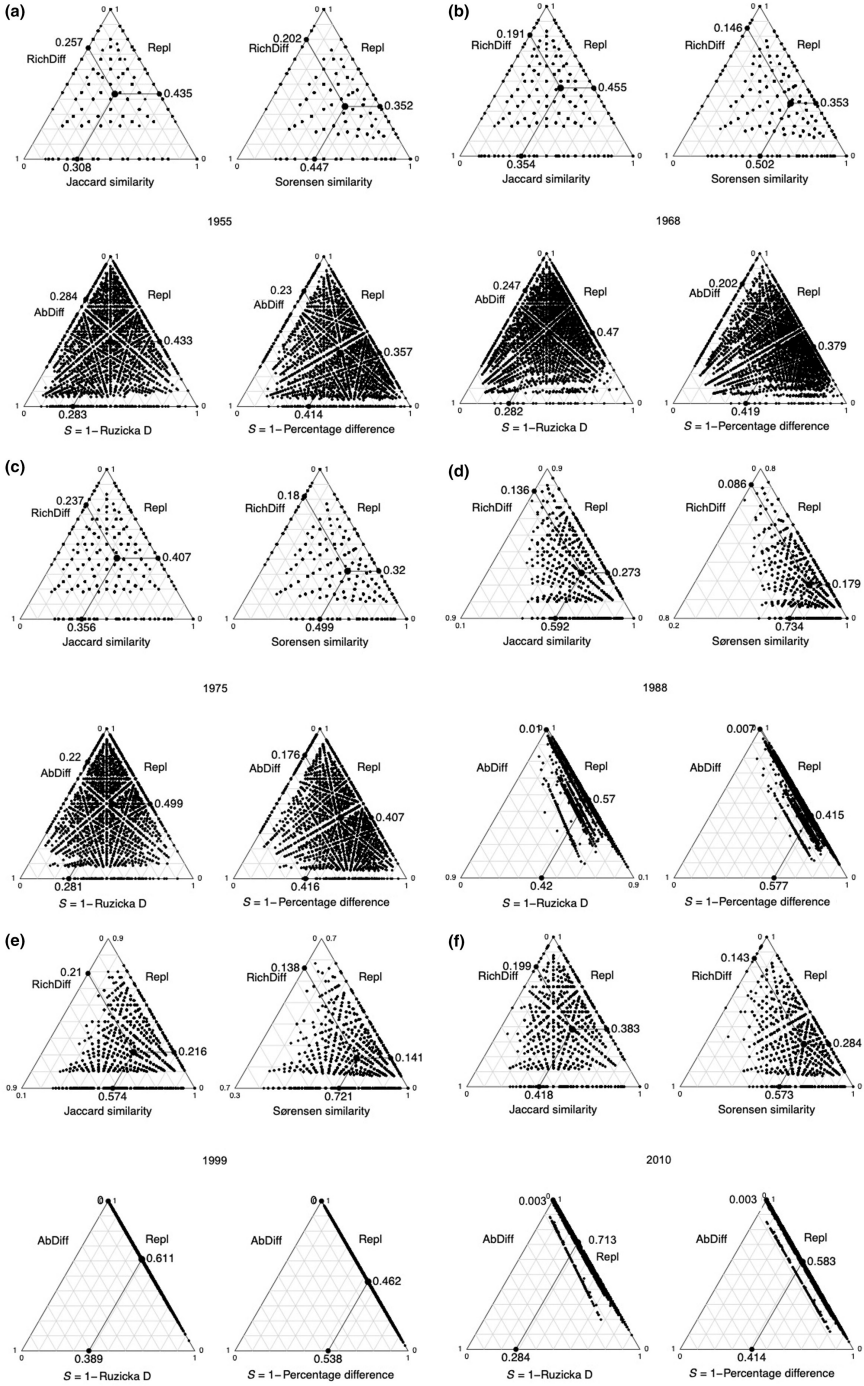
Simplex plots, following Podani and Schmera (2011) for (a) 1955, (b) 1968, (c) 1975, (d) 1988, (e) 1995, and (f) 2010. We used four indices of β diversity (Jaccard, Sørensen, Ružička, and percentage difference). The Ružička and percentage difference indices are quantitative equivalents of the Jaccard and Sørensen qualitative indices, with the Sørensen index being weighted twice as heavily as the Jaccard index (see Section 2). AbDiff, absolute difference; Repl, replacement; RichDiff, richness difference.

In the first 40 years, we found no significant changes in plots (LCBD), regardless of whether the plots were assessed by Jaccard, Sørensen, Ružička, or percentage difference. However, there was a slight change among a few plots in 1988, followed by a substantial change in many plots in 1995 and 2010 (Figure 3). The major changes were in terms of species replacement and not in species differences (or absolute differences in the cases of Ružička or percentage differences), which were very close to 0.

In 1955, 1968, and 1975, there were no significant changes in species presence (SCBD), based on Holm's correction of *p* values. In 1988, 1995, and 2010, there were up to 17 species that had significantly higher values than the mean contribution of species to β diversity (SCBD; Table 1). There were eight species that had significantly higher values than the mean SCBD in all three census years: *Aristida junciformis*, *Cymbopogon caesius* (formerly *C. excavatus*), *Diheteropogon amplectens*, *Eragrostis curvula*, *E. racemosa*, *Heteropogon contortus*, *Themeda triandra*, and *Tristachya leucothrix* (Table 1).

**TABLE 1:** Species that had significantly higher SCBD values than the mean SCBD across years, based on the Holm correction of *p* values.

Species	1988	1995	2010
*Aristida junciformis* Trin. & Rupr.	*	*	*
*Cymbopogon caesius* (Hook. & Arn.) Stapf	*	*	*
*Cymbopogon nardus* (L.) Rendle			*
*Diheteropogon amplectens* (Nees) Clayton	*	*	*
*Elionurus muticus* (Spreng.) Kuntze	*		
*Eragrostis capensis* (Thunb.) Trin.	*	*	
*Eragrostis curvula* (Schrad.) Nees	*	*	*
*Eragrostis racemosa* (Thunb.) Steud.	*	*	*
*Heteropogon contortus* (L.) Roem. & Schult.	*	*	*
*Hyparrhenia hirta* (L.) Stapf	*		*
*Melinis nerviglumis* (Franch.) Zizka	*		
*Microchloa caffra* Nees	*		
*Panicum aequinerve* Nees			*
*Sporobolus africanus* (Poir.) Robyns & Tournay			*
*Setaria nigrirostris* (Nees) T. Durand & Schinz	*		
*Themeda triandra* Forssk.	*	*	*
*Tristachya leucothrix* Trin. ex Nees	*	*	*

*Note*: There were no significant changes in 1955, 1968, and 1975. * indicate significant effect.

### 3.1 Beta regression

There was no significant relationship between the Jaccard (presence–absence) index of β diversity and the three explanatory variables, with the exception of the intercept (phi ± SE = 23.443 ± 2.829, *z* = 8.287, *p* < .001). The maximum likelihood pseudo‐*R* ^2^ for the Jaccard index of β diversity was .01346. However, there was a significant relationship between the Bray–Curtis (percentage differences) index and the explanatory variables for the main effects (estimate ± SE = 25.095 ± 3.031, *z* = 8.28; *p* < .001) (Table 2). The pseudo‐*R* ^2^ was .1408 (*p* < .001). The relationship between the Bray–Curtis (quantitative) index and the explanatory variables for the main effects and the mowing*burning frequency interaction had a pseudo‐*R* ^2^ value of .144 (*p* < .001). There was no significant difference between the main effects only and the main + interaction effects (likelihood ratio test: *χ* ^2^ = 0.504, *p* = .4777). The only significant explanatory variable was mowing (Table 2).

**TABLE 2:** β regression of mowing (spring, summer, and spring + summer), time of burn, and burn frequency (annual, biennial, triennial).

Variable	Estimate	SE	*z* Value	Pr(>|*z*|)
Pseudo‐*R* ^2^ = .1408; *p* < .001				
(Intercept)	4.424e‐02	1.109e‐01	0.399	.690
Burn frequency	2.577e‐05	3.039e‐02	0.001	.999
Burn time	6.459e‐03	1.194e‐02	0.541	.589
Mow	1.533e‐01	3.231e‐02	4.746	<.001***
Pseudo‐*R* ^2^ = .1440; *p* < .001				
Intercept	−0.007834	0.133257	−0.059	.95312
BurnFreq	0.032504	0.054850	0.593	.55344
BurnTime	0.005958	0.011945	0.499	.61793
Mow	0.187741	0.058565	3.206	.00135**
BurnFreq:Mow	−0.020516	0.029073	−0.706	.48040

*Note*: We used a mean model with logit link. The pseudo‐*R* ^2^ with main effects only (.1408) was very similar to that with interaction effects (.144). The only significant predictor variable in both comparisons was mowing. ** indicate significant, p < .05; *** indicate significant, p < .001.

When we included the effect of soil depth in each plot, we found that there was a significant interaction effect between mow frequency and soil depth, but there were no other significant main or interaction effects (Table 3). Significantly more variance was explained by including soil depth (pseudo‐*R* ^2^ was .39).

**TABLE 3:** Plot analysis of the β regression including mowing frequency (Mow: spring, summer, and spring + summer), time of burn (BurnTime: monthly), and burn frequency (BurnFreq: annual, biennial, triennial) and soil depth at the plot level.

Coefficients	Estimate	SE	*z* Value	Pr(>|*z*|)
(Intercept)	−0.0315558	0.2470052	−0.128	.898344
BurnFreq	0.0294117	0.1345932	0.219	.827022
Mow	−0.1097666	0.1017668	−1.079	.280762
BurnTime	−0.0001621	0.0316530	−0.005	.995913
Soil_Depth	0.0015795	0.0066721	0.237	.812862
BurnFreq:Mow	−0.0153369	0.0621254	−0.247	.805009
BurnFreq:BurnTime	0.0027421	0.0151846	0.181	.856694
Mow:BurnTime	0.0127596	0.0115143	1.108	.267794
Mow:Soil_Depth	0.0091102	0.0024174	3.769	.000164***
BurnFreq:Soil_Depth	0.0002257	0.0021882	0.103	.917847
BurnTimeNum:Soil_Depth	−0.0002067	0.0007859	−0.263	.792582
BurnFreq:Mow:BurnTime	−0.0036187	0.0081848	−0.442	.658400

*Note*: We used a mean model with logit link. The pseudo‐*R* ^2^ was 0.39. The sole significant predictor was mowing by soil depth. *** indicate significant, p < .001.

### 3.2 Distance‐based redundancy analysis (dbRDA)

We ran a multivariate distance‐based redundancy analysis for 2010. There was no significant effect of richness difference (*F* = 0.255, *p* = .90, error df = 128, *n* = 999 permutations). However, we found that there was a significant effect of replacement (*F* = 10.832, *p* < .001, error df = 128, *n* = 999 permutations).

**FIGURE 4:**
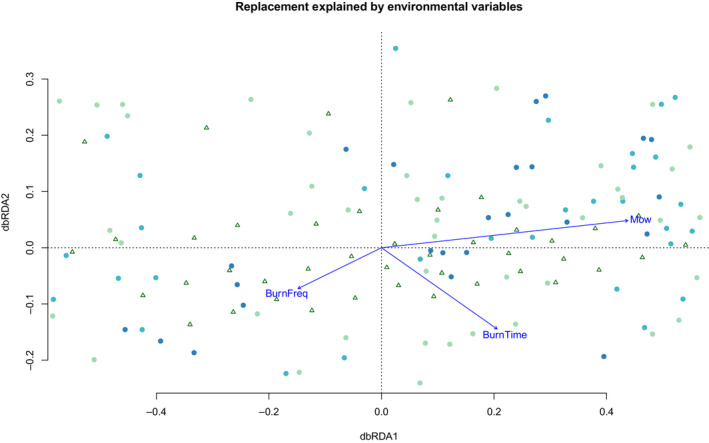
Distance‐based RDA for 2010 for replacement differences based on the environment variables, Burning Frequency (unburnt, annual, biennial, triennial), Burning Time (unburnt, winter, spring, or autumn), and Mowing Frequency (unmowed, mowed in Spring, mowed in Summer, and mowed twice (Spring and Summer)). The darker the circle, the more frequently it was mowed (i.e., spring and summer each year). Triangles = unmowed.

The proportion of the constrained variance (“partitioning of squared unknown distance”) was 20.25%, and the unconstrained variance was 79.75% for replacement. The first dbRDA axis for replacement explained 87.78% of the variance (eigenvalue = 4.4641), followed by 8.82% of the variance for dbRDA2 (eigenvalue = 0.4486). There was a significantly adjusted *r* ^2^ value for replacement (*r* ^2^ = .18, *p* < .001). Based on the dbRDA1 biplot score for constraining variables, most of the variance was explained by mowing frequency (dbRDA1 = 0.8664), followed by burn time (0.4064), and burn frequency (−0.2936). On the 2nd dbRDA axis, burn time explained most of the variance (−0.9021), followed by burn frequency (−0.4538), and then by mowing frequency (0.3016) (Figure 4). There was only a slight increase in variance that was explained by Soil Depth (adjusted *r* ^2^ = .21, *F* = 8.491, *p* = .001). The direction of the effect of Soil Depth was very close to that of Burn Frequency and opposite that of Mowing.

## 4 DISCUSSION

Turner et al. (2003), Borer et al. (2014), and Farley et al. (2018), among others, have stressed the importance of large‐scale experiments and replication of experiments within a particular ecosystem (such as grasslands, in this case) as being particularly valuable for understanding the role of successional change among different treatments over extended periods. One of the key values demonstrated in the current experiment was the elucidation of the role of β diversity in this subtropical grassland. We found that most of the variance in grass β diversity was due to replacement in time rather than richness differences, as we predicted (Prediction 1). This result is consistent with some other studies (e.g., Jones et al., 2017; Seabloom et al., 2020; Weisser et al., 2017) but definitely not all (Atmar & Patterson, 1993; Baselga, 2012; Legendre, 2014; Soininen et al., 2018). An earlier study by Fynn and O'Connor (2005) at Ukulinga also showed that changes were largely due to the replacement of species. This is not to say that β diversity at Ukulinga did not change over time. Indeed, there were lots of changes in species composition over time (Fynn et al., 2005; Titshall et al., 2000; Ward et al., 2020; Ward, Kirkman, & Tsvuura, 2017), but this did not significantly affect β diversity. For example, at Ukulinga, *Harpochloa falx* was common at the beginning of this experiment in 1950 but disappeared by 1955. *Eragrostis curvula* appeared in 1955 (but not prior to that) and increased steadily throughout, reaching 8% in the BME in 2010. *Cymbopogon nardus* was absent until 1968 and increased in relative abundance to a value of 8% by 2010. Indeed, *C. nardus* became the dominant species in triennial burns (Ward et al., 2020). Other prominent changes in species composition were the undefoliated plots (unburned and unmowed treatments), where many trees, shrubs, and two herbaceous plants appeared, including several invasive species (Titshall et al., 2000), such as *Acacia melanoxylon* (Australia), *Jacaranda mimosifolia* (south‐central South America), *Melia azedarach* (Indonesia‐Malaysia), *Lantana camara* (American tropics), *Tagetes minuta* (herbaceous; southern half of South America), *Solanum mauritianum* (South America), *Bidens pilosa* (herbaceous; Americas), as well as some indigenous encroaching trees (*Acacia* (*Vachellia*) *nilotica*, *Acacia* (*Vachellia*) *karroo, Lippia javanica*, and *Dichrostachys cinerea*). Morris and Tainton (2002), Tsvuura and Kirkman (2013), and Ward et al. (2020) noted that the grass *Aristida junciformis* also became dominant in the undefoliated plots, and those burnt at lower frequency. Previously, *A. junciformis* was dominated by *Themeda triandra* and *Heteropogon contortus* in these plots (Morris & Tainton, 2002).

Inconsistent with our second prediction, we found that changes in β diversity were ostensibly slow, and were explicit only from 1988 onwards (i.e., 38 years after the start of the experiment), despite the fact that subtropical ecosystems might respond more rapidly than temperate ecosystems (Ward et al., 2020). We note that measuring basal cover, as was done in the period prior to 1988, can be imprecise without a very large sample size, especially for less abundant species (Mentis et al., 1980). The resultant species‐abundance data using 200‐point hits can be sparse, typically with fewer than 20 species incidences per plot (<10% hits). Consequently, the absolute‐cover abundance data or proportions relativized by (low) plot totals may be imperfect. Estimates of grass species richness per plot are less reliable because the number of species encountered in the older surveys was low (median ~ 5; Mentis et al., 1980). Grass species richness doubled when nearest‐plant data were collected from 1988 onwards (Table 4). The same pattern was detected with the SCBD (Table 1), where no significant changes occurred prior to 1988. Such changes could have been due to increased α diversity in individual plots, especially in rare species, from 1988 onwards. Mentis (1981) and Short and Morris (2016) recorded almost no bias when using the nearest‐plant method for determining species composition. We conclude that the power to detect treatment effects in the imprecise point‐hits data may be low in the older surveys, and increased when nearest‐plant data were used. Therefore, any differences over time could be due to a switch in methodology, from estimating species cover to species frequency, and not a real change. Unfortunately, we do not have a reliable way of differentiating these. We recognize that there could alternatively have been increases in species richness with time, much in the same way as described by Arnillas and Cadotte (2019) and Arnillas et al. (2022; see below).

**TABLE 4:** Summary statistics for data sampled in different years at Ukulinga, using two different techniques, point hits and nearest plant (see Section 2 for further details).

Year	1955		1968		1975		1988		1995		2010^a^	
Method	Point hits		Point hits		Point hits		Nearest plant		Nearest plant		Nearest plant	
	Plot total	Species richness	Plot total	Species richness	Plot total	Species richness	Plot total	Species richness	Plot total	Species richness	Plot total	Species richness
Minimum	0	0	4	1	4	2	75	8	100	5	86	4
Maximum	30	8	28	9	24	10	116	19	200	19	100	16
Median	12	4	17	6	13	5	100	13	200	13	100	10
Mean	12.2	4.0	17.1	5.8	13.0	5.3	99.8	12.9	199.2	12.6	99.7	9.6

*Note*: *Plot Total* indicates the number of individual plants recorded.

^a^ Some individual non‐grass (forb and tree) species in addition to the lumped “forb” category.

Regarding changes in species, significant changes were also only recorded in the final three periods. *Themeda triandra*, *Tristachya leucothrix*, and *Aristida junciformis* recorded significant changes over the three last‐mentioned periods (1988, 1995, 2010). *Diheteropogon amplectens*, *Eragrostis curvula*, *E. racemosa*, and *Heteropogon contortus* also changed in the last three periods. *Cymbopogon nardus* responded most strongly to triennial burns in 2010 but not prior to that (Table 1). This slow change in replacement in the subtropical grassland is surprising in terms of the rapid rates of grassland changes recorded in many studies (Dembicz et al., 2021; Eek & Zobel, 2001; Lepš, 2014). Indeed, some grassland studies have shown that replacement can occur over a decade or less (e.g., Avolio et al., 2014; Balogianni et al., 2014; Ceballos et al., 2010; Török et al., 2010). However, Weisser et al. (2017) also found that the replacement of species in a grassland in Jena in Germany was similarly slow. Similarly, Seabloom et al. (2020) found that the recovery of grassland ecosystems was slow at Cedar Creek, Minnesota, USA.

Inconsistent with our third prediction, β diversity did not respond more to burning frequency and timing than to summer mowing frequency (Ward, Kirkman, Hagenah, & Tzvuura, 2017), despite the fact that burning is often considered to be most important for maintaining tall grasslands in the absence of summer mowing such as at Ukulinga or with selective livestock or wild ungulate defoliation (Bond, 2005; Bond et al., 2005; Fynn et al., 2004; Kirkman et al., 2014; Morris et al., 1992; Morris & Tainton, 2002; Tainton et al., 1978). Frequent application of burning (biennially) is often prescribed for areas where summer defoliation is absent (such as in wildflower reserves where mammalian herbivory may be absent) or not sufficiently intense to reduce grass biomass in productive years, and after long‐rest periods (Brown et al., 2016; Carbutt et al., 2017; Govender et al., 2006). Both the β regression (Bray–Curtis index only) and the multivariate dbRDA showed that mowing was the most important factor, with β regression demonstrating that only mowing was significant. We also found a significant interaction effect between mowing frequency and soil depth on a per‐plot basis (Table 3).

When there were better‐growing conditions in deeper soils that allowed trees to grow (and mowing frequency = 0), this resulted in altered grass composition and diversity. For example, tall grasses, such as *Cymbopogon* and *Hyparrhenia* species, were more abundant on deeper soils in Block 3 (Figure 2) and under no mowing or combined with winter or spring mows (Table S1); these large grasses outcompeted subordinates and reduced grass diversity (thereby reducing ß diversity). In the dbRDA study, there was also an effect of burning frequency and burn time, but mowing was the most important factor. This is in contrast to our earlier finding that there was a significant interaction effect between fire and mowing on species richness, with the effect of fire being dominant (Ward et al., 2020). In our earlier studies, mowing resulted in a decline in species richness as well as a decline in aboveground yield (Ward, Kirkman, Hagenah, & Tzvuura, 2017; Ward et al., 2020; Figure 3). Mowing is not prescribed to maintain grassland because it is costly and impractical in rugged terrain. Burning could be essential to maintain fire‐dependent forbs (and thus diversity; Fynn et al., 2004; Uys et al., 2004), but our study did not examine that factor because forbs were rare. Contrastingly, in the Great Plains of the U.S., Dickson et al. (2019) found that annual burns in autumn resulted in an increased abundance of forbs, but spring and summer burns did not. Morris et al. (2021) found that frequent burning maintained a stable grassland over 40 years in the mountains of KwaZulu‐Natal, South Africa. In a comparison of mowing and burning as removal treatments in the KwaZulu‐Natal Tall Grassland, mowing proved to be superior to burning in terms of sward productivity (i.e., in terms of Aboveground Net Primary Productivity (ANPP)) in the season following the removal treatment, but burning was responsible for a sward of higher protein content, at least during the early part of the season (Tainton et al., 1977). Similarly, Mbatha and Ward (2010) found that fire in interaction with fertilizer and grazing resulted in an increase in protein concentration. In our previous study, a similar result for ANPP was also obtained, with the exception of the control plots, where many exotic species invaded (Ward et al., 2020). We found that species richness declined across mowing treatments in our earlier study (Ward et al., 2020), but there were no changes in species richness between intermediate and infrequent burns, unlike the situation reported for Konza Prairie (Collins et al., 1998; Knapp et al., 1998). Dominance by a few species occurred at Ukulinga and in Konza Prairie and Park Grass (England) experiments under high productivity (such as Ward, Kirkman, & Tsvuura, 2017) and frequent burns. Annual burns resulted in lower richness at both Ukulinga and Konza Prairie, but there was no significant difference in species richness among biennially and triennially burned plots at both sites (Ward et al., 2020). McGranahan et al. (2018) found in temperate Great Plains grasslands in Oklahoma that the greatest β diversity among plant functional groups occurred in landscapes with three to four patches (25%–33%) of area burned and three‐ to four‐year fire return intervals.

The predominant effect of summer mowing frequency (particularly zero mow versus one or two mows) is inconsistent with several previous studies, including a meta‐analysis, that indicated that mown sites supported a greater number of plant species than sites that were either grazed, or grazed and mown (Chen et al., 2021; Zhu et al., 2020, 2021). Zhu et al.'s (2020, 2021) studies were meta‐analyses in temperate grasslands in northern China whereas ours was a subtropical grassland. Whether the difference was due to the climate is unclear (Abbadie et al., 2006; Gibson, 2009; Smith et al., 2016; Wilsey, 2018). The effects of mowing frequency were demonstrated by an early study by Tainton and Booysen (1965) at Ukulinga, who found that clipping twice per year (once early and once late) to simulate mowing reduced the weight of primary tillers at maturity of the predominant *Themeda triandra* at Ukulinga by 14% when compared with no clipping in the following summer. Coughenour et al. (1985) showed in the tropical Serengeti that clipping reduced most plant yield components. After 2 months, the leaf elongation rate was greater in clipped plants in the Serengeti, but over the whole experiment, aboveground yield was unaffected by clipping. Similarly, in the humid subtropical Lamto savanna of the Cote d'Ivoire (West Africa), Leriche et al. (2003) found that there was a significant decline in ANPP with increased clipping intensity. These last‐mentioned authors also found that nitrogen concentration increased with repeated clipping. We note that Socher et al. (2012, 2013) found that there was a substantial effect of mowing in several temperate sites in Germany, which exceeded the effects of grazing and fertilization. In Argentina, Molina et al. (2020) found that mowing did not affect the negative impact of fertilization on species richness. However, we did not include fertilization or grazing in this study. Klimešová et al. (2008) have suggested that different traits should be measured to assess the effects of mowing and burning. In addition to plant height, Klimešová et al. (2008) suggested that factors such as clonal architecture (see also Baer et al., 2016), the bud bank, and nonstructural carbohydrate storage would be more effective in assessing the effects of mowing and burning.

In our study, there was a significant effect of soil depth on grass β diversity from our β regression (Table 3), but the difference was very slight in the multivariate analysis (dbRDA). Regardless, there was a lower effect than the effects of mowing frequency. Contrastingly, Braun et al. (2022) found that there was a strong positive association with mowing, whereas we found that the effect of soil depth was negative. Nonetheless, more β diversity was explained in our study by incorporating soil depth than by excluding it, particularly in the β regression. Thus, our results differed between the classical (β regression) and multivariate analyses, with considerably more variance being explained by the β regression *contra* the claims of Bennett and Gilbert (2015).

The environmental heterogeneity hypothesis (EHH) could also partially explain the effects of soil depth, with variance in soil depth being one of the parameters considered (Baer et al., 2016). The EHH predicted that there would be greater species coexistence in sites with greater EHH. However, Baer et al. (2016) found that there was a diminished effect of environmental heterogeneity in their study at Konza Prairie (Kansas, USA) and that this was counter‐balanced by the effect of a dominant clonal grass, *Andropogon gerardi*. They claimed that high biodiversity may depend more on reducing the dominance of a few species and dispersal of new species (i.e., increased species richness difference) than on promoting environmental heterogeneity per se. In our study, soil depth was not unduly convincing as an important variable controlling β diversity, and only had an effect as an interaction with mowing, although we must concede the topography at Ukulinga was slight.

### 4.1 Possible phylogenetic constraints

We excluded phylogenetic constraints on β diversity (*contra* Gerhold et al., 2015, 2018) that imply that certain species are less likely to occur because of their phylogenetic similarity to other species (Cadotte et al., 2009, 2017; Forrestel et al., 2014, 2017). At Cedar Creek (Minnesota), phylogenetic diversity, along with the presence of a nitrogen fixer and seed mass were better explanations for community productivity than seven other alternative models that included species richness, functional traits, and functional group richness (Cadotte et al., 2009). Similarly, Gerhold et al. (2015, 2018) have shown that phylogenetic explanations can be more effective than other similarity indices. However, in their study, there was the confounding factor that phylogenetic differentiation is based on the fact that nitrogen‐fixing plants are mostly in a single clade, the legumes (Fabaceae), leading to an exaggerated representation of these species (Davies et al., 2016; Roscher et al., 2011). Cadotte et al. (2017) and Davies (2021) note that inappropriate phylogenies, skewed distributions of phylogenetic distances, the lack of consideration of models of trait evolution, or the absence of sufficient niche space in experimental and observational venues could also hamper the role of phylogenetics in community assembly. Our study focused largely on grasses (Poaceae; i.e., excluding nitrogen‐fixing species). Furthermore, all of these species were C_4_ perennial species, further potentially reducing the variability explained by phylogeny. Importantly, this was an applied study, so we focused primarily on the disturbance effects of mowing and burning, and did not consider whether phylogeny was also involved.

We recognize, however, that some of the variance can be explained by phylogeny. For example, Arnillas and Cadotte (2019) found that dominant plants moderated the environment where nondominant species thrived, diminished the influence of environmental filtering, and increased the influence of limiting similarity for nondominant species. Contrastingly, in a meta‐analysis of 78 grasslands across five continents, Arnillas et al. (2022) showed that dominant species were phylogenetically clustered, indicating that there were co‐dominant grasses. This indicated that the dominant species were organized by environmental filtering (and not by competition), and that nondominants were either randomly assembled or overdispersed (but see Kraft et al. (2015) for an alternative view). In a study performed at Ukulinga and Konza Prairie, Forrestel et al. (2014, 2017) discovered effects related to fire that were due in part to the phylogenetic relatedness of these species at Ukulinga and Konza Prairie. In both sites, Forrestel et al. (2014) found that there was phylogenetic clustering of highly abundant species in annually burned and less frequently burned plots, driven by species of the Andropogoneae. They also found significant effects in the unburned plots at Ukulinga but not in the unburned plots at Konza Prairie. Forrestel et al. (2014) did not examine the mowed plots.

## 5 CONCLUSIONS

Our study showed that changes in grass β diversity were almost entirely explained by replacement, rather than species richness differences. Furthermore, change in this subtropical grassland ecosystem was apparently slow; changes were only manifest from 1988 onwards. This is the first study to specifically examine β diversity at Ukulinga. All previous studies on this experiment have drawn conclusions based on treatment effects on species composition (i.e., mostly they dealt with α diversity; e.g., Kirkman et al., 2014; Morris et al., 1992; Tsvuura & Kirkman, 2013; Ward et al., 2020; Ward, Kirkman, Hagenah, & Tzvuura, 2017). Such studies are complementary because they dealt with different aspects of diversity (Smith et al., 2016). Studies from this ecosystem that indicated significant β diversity changes, such as in mowing and burning, that occurred earlier than 1988 can be considered to be premature (e.g., Tainton et al., 1977, 1978), but others were not (e.g., Fynn et al., 2004, 2005, 2011; Fynn & O'Connor, 2005; Kirkman et al., 2014; Tsvuura & Kirkman, 2013; Ward et al., 2020; Ward, Kirkman, Hagenah, & Tzvuura, 2017; Ward, Kirkman, & Tsvuura, 2017). Nonetheless, changes in net primary production could have been important (Fynn et al., 2005; Morris & Tainton, 2002; Tainton et al., 1977, 1978). Indeed, Morris and Tainton (2002) have suggested that, at Ukulinga, at least 10 or even as much as 20 years were required to assess the effects of burning on grass species composition. Studies in other countries that proclaimed rapid changes (e.g., Braun et al., 2022; Ceballos et al., 2010; Török et al., 2010) should look perhaps at extending these studies to ascertain whether claims of rapid changes could be justified by β diversity studies as indicated here.

## AUTHOR CONTRIBUTIONS

**David Ward:** Conceptualization (equal); data curation (equal); formal analysis (equal); funding acquisition (lead); investigation (lead); methodology (lead); project administration (lead); resources (lead); software (lead); supervision (equal); validation (equal); visualization (lead); writing – original draft (lead); writing – review and editing (supporting). **Kevin Kirkman:** Conceptualization (equal); data curation (equal); formal analysis (supporting); funding acquisition (supporting); investigation (supporting); methodology (supporting); project administration (supporting). **Craig Morris:** Conceptualization (supporting); data curation (equal); formal analysis (supporting); funding acquisition (supporting); investigation (supporting); methodology (supporting); project administration (supporting); resources (supporting); software (supporting); supervision (supporting); validation (supporting); visualization (supporting); writing – original draft (supporting); writing – review and editing (equal).

## Supporting information


Table S1.

## Data Availability

Our Supplementary file is available on OAKS at https://doi.org/10.21038/ward.2023.0601. Once subsequent analyses are completed, data shall be made available via DRYAD (KK).
