# Supplementary material for: Long‐term subtropical grassland plots take a long time to change: Replacement is more important than richness differences for beta diversity
Source: Ecol Evol. 2023 Jun 14;13(6):10.1002/ece3.10195. doi: 10.1002/ece3.10195 (PMC10266706; doi:10.1002/ece3.10195)
Supplement: Supplementary file 1 — Table S1. [file ECE3-13--s001.docx]

**Supplementary Files**

**Supplementary Table 1.** Burning and mowing experimental design. All plots were replicated three times. The entire plot was mowed and burned in whole plots B-D and sub-plots 2-9. A1 were complete controls, i.e. no mowing and no burning. Note that sub-plots 10 and 11 are mows and not burns.

**Whole-plot**

**A** Control (not cut for hay)

**B** One hay cut early summer when grass is ca. 200 mm high

**C** One hay cut late in the summer (end of February)

**D** Two hay cuts, one early (B) and one late (C) summer

**Sub-plot**

**1** Control (no burn)

**2** Annual burn first week of August

**3** Annual burn after first effective spring rains (12.5 mm in 24 h)

**4** Biennial burn first week of August

**5** Biennial burn after first effective spring rains (12.5 mm in 24 h)

**6** Biennial burn in autumn (mid-May)

**7** Triennial burn first week of August

**8** Triennial burn after first effective spring rains (12.5 mm in 24 h)

**9** Triennial burn in autumn (mid-May)

**10** Annual *mow* in first week of August

**11** Annual *mow* after first effective spring rains (12.5 mm in 24 h)
